# Reading-impaired children improve through text-fading training: analyses of comprehension, orthographic knowledge, and RAN

**DOI:** 10.1007/s11881-021-00229-x

**Published:** 2021-05-11

**Authors:** Telse Nagler, Jelena Zarić, Fenke Kachisi, Sven Lindberg, Jan-Henning Ehm

**Affiliations:** 1 DIPF, Leibniz Institute for Research and Information in Education, Rostocker Str. 6, 60323 Frankfurt, Germany; 2 Center for Individual Development and Adaptive Education of Children at Risk (IDeA), Frankfurt, Germany; 3 Department of Clinical Developmental Psychology, University of Paderborn, Paderborn, Germany

**Keywords:** Orthographic knowledge, RAN, Reading comprehension, Reading-impaired children,, Reading rate, Text-fading training

## Abstract

Early intervention for children with reading impairments is crucial in order to achieve reading improvements and avoid school failure. One line of reading intervention research focuses on the experimental manipulation of reading rate through a text-fading training approach. Considering relevant reading-related predictors (i.e., orthographic knowledge and rapid automatized naming; RAN), we aim at evaluating the text-fading training’s efficiency for a sample of German reading-impaired third graders (*n* = 120). The purpose of the present study was to examine (1) the predictive value of orthographic knowledge and RAN and their contribution of explained variance in comprehension performance during training, (2) text-fading training effects on reading rate and comprehension in a pre-post comparison, and (3) (lasting) text-fading training effects at word and sentence level in a pre-post-follow-up design. Results of structural models indicated RAN to be significantly related to comprehension performance for the experimental group, whereas no sufficient regression weight was found for orthographic knowledge. A reverse pattern was found for the self-paced group. No significant improvements regarding reading rate and comprehension were revealed for the experimental group after training. However, significant positive effects on word and sentence level at post-test time point indicate stronger reading improvements for the experimental compared to the control group. The retention of training gains was indicated at sentence-level reading 6 months after the training. Possible explanations for the presented positive training effects as well as the mixed results for reading rate, comprehension, and follow-up preservation are discussed.

Most children successfully master the acquisition of complex reading processes during elementary school, through formal education and reading practice (Huemer et al., 2008). Practice and exposure to print are considered to foster more automatic processing, which enables the reader to identify and coordinate words into sentences without paying much attention to this process. Thus, automatized word recognition leads to increased reading speed and reading fluency, often defined as fast and effortless decoding with correct prosody and a shift of attention to higher processes (Wolf & Katzir-Cohen, 2001). Increased reading fluency is strongly associated with improved reading comprehension (Huemer et al., 2008), which is considered the ultimate goal of reading (Pennington, 2009). It comprises accessing and constructing meaning from written text and coordinating multiple levels of language and cognitive functions (Johnston et al., 2008; Landi & Ryherd, 2017). However, about 4–9% of children show significant reading acquisition difficulties (Moll et al., 2014). Empirical evidence supports the notion that especially deficits in reading fluency characterized by slow and laborious reading performance (de Jong & van der Leij, 2003) are a major component of reading impairments (e.g., Kuhn & Stahl, 2003; Ziegler et al., 2003). Impaired reading is closely related to problematic reading comprehension performance (Landerl & Wimmer, 2008). Different reading-relevant precursors, such as the automatized and fast retrieval of information (i.e., rapid automatized naming (RAN)) and the orthographic knowledge, defined as the quality of orthographic representations allowing access to linguistic information stored in the mental lexicon, affect successful reading development, and therefore reading comprehension (Catts et al., 2015), as well as reading intervention outcome.

Obviously, children with reading impairments are in special need for reading intervention, which should be introduced as early as possible, best during elementary school years (Brassel & Rasinski, 2008; Potocki et al., 2015), to improve reading skills and prevent school failure. The purpose of the present study is to evaluate the efficiency of a reading fluency training approach (i.e., text-fading training) with specific consideration of relevant reading-related predictors. More precisely, we firstly aim at examining the predictive value of orthographic knowledge and RAN and their contribution of explained variance in comprehension performance during training. Secondly, we investigate text-fading training effects on reading rate and comprehension performance in a pre-post comparison. Thirdly, we engage in analyzing (lasting) text-fading training effects at word and sentence level in a pre-post-follow-up design after controlling for predictive reading-related variables.

## Lower- and higher-level reading processes

With regard to the complexity of the reading process, it is important to distinguish between different levels and preconditions necessary for successful reading development and reading intervention. Particularly, the structure and nature of the reading material has to be considered. Single word reading, which can be acknowledged as the basis for connected text reading, requires mostly orthographic and phonological skills that are considered lower-level processing (Wolf & Katzir-Cohen, 2001). For connected text reading, such as sentence reading, language processing and comprehension skills are also necessary (Jenkins et al., 2003), which are considered to be higher-level reading processes. Furthermore, to comprehend more complex reading material, an efficient reading rate is necessary (Kuhn & Stahl, 2003). Considering the interplay of lower-level and higher-level reading processes with the bi-directional interaction of decoding, reading comprehension, and reading rate, it is essential to have a closer look at relevant reading-related subskills. In this regard, especially orthographic knowledge as well as RAN have been identified as highly predictive for reading operations (Loveall et al., 2013).

## Orthographic knowledge and rapid automatized naming as reading predictors

Orthographic knowledge comprises the knowledge of conventions and rules about a specific orthography (Conrad et al., 2013). There is a consensus that orthographic knowledge consists of two components: a word-specific component containing the knowledge about the spelling of specific words and a general component containing the knowledge about permitted letter patterns of a written language (Apel, 2011; Rothe et al., 2015). Fluent reading is supported by a sufficient level of orthographic knowledge, enabling the individual to recognize written letter patterns as words with little cognitive effort (e.g., Ehri, 2005, 2014). Empirical research has shown that orthographic processing predicts reading fluency in different languages, such as English and Greek (Georgiou et al., 2008). Furthermore, both subtypes of orthographic knowledge have been revealed as significant predictors for lower-level reading, such as word reading accuracy and speed, in different orthographies varying in the transparency level, indicating that lower reading performance is associated with fewer orthographic knowledge skills (e.g., English: Deacon et al., 2013; Greek: Georgiou, Parrila, & Papadopoulos, 2008b; German: Rothe et al., 2015; Zarić, Hasselhorn, & Nagler, 2021; Zarić & Nagler, 2021). Additionally, both word-specific and general orthographic knowledge have been identified to be relevant also for higher-level reading performance, such as sentence (Katzir et al., 2006; Zarić & Nagler, 2021) and text comprehension (Zarić et al., 2021).

RAN is characterized by fast and automatized naming of known stimuli, such as digits, letters, objects, and colors (Denckla & Rudel, 1976). According to Bowers and Newby-Clark (2002) and Bowers and Wolf (1993), it represents the general processes of cognitive speed. Empirical research studies suggest RAN to be a timing mechanism reflecting the speed of retrieval of phonological information as well as the formation of orthographic patterns and their integration with phonological codes (Manis et al., 1999; Mayringer & Wimmer, 2000). RAN is suggested to reflect the ability to recognize adjacent letters, thus, allowing their chunking into familiar letter patterns. Therefore, it is assumed that RAN is closely connected to the acquisition of orthographic knowledge, and hence, to contribute to efficient reading (Bowers & Newby-Clark, 2002; Wolf & Bowers, 1999). A large body of research shows that RAN is a significant predictor for reading skills (e.g., English: Georgiou, Das, & Hayward, 2008a; McCallum et al., 2006; English/Greek: Georgiou et al., 2008; German: Landerl & Wimmer, 2008; Moll et al., 2009) and among the most robust correlates of reading impairments (Landerl et al., 2013). A meta-analysis by Araújo et al. (2015) furthermore indicates that RAN is significantly associated with several different reading sub-components on lower-level (i.e., word reading, pseudo-word reading) as well as higher-level (i.e., reading comprehension) processing.

Besides the predictive value of orthographic knowledge and RAN for reading acquisition and reading difficulties, several studies have pointed out that both components can significantly influence intervention success. In a meta-analysis from Nelson et al. (2003) for example, the responsiveness to reading interventions was more strongly associated with RAN than with other cognitive components, such as phonological awareness or phonological memory. Similarly, orthographic skills were shown to differ between reading intervention outcomes (Berninger et al., 1999). Orthographic skills and naming speed were shown to predict the improvement of word reading performance after intervention (Scheltinga et al., 2010; Stage et al., 2003). Consequently, orthographic knowledge and RAN should be considered predictors when measuring training efficacy and possible training effects.

## Reading fluency intervention approach: text-fading training

One approach to supporting children with impaired reading performance is to increase reading fluency through training. Reading fluency trainings are manifold, and reading rate as well as reading comprehension have been manipulated in several ways. Most often, reading rate has been treated as an outcome variable depending on effective word recognition and comprehension (Carver, 1990; Gough & Tunmer, 1986). The specific research line presented in the following, however, suggests considering individual reading rates as an independent and experimentally manipulable variable (Breznitz & Berman, 2003).

### Text-fading manipulation

The text-fading approach is based on a text-fading procedure manipulating the rate in which reading material is faded out from a computer screen letter by letter in reading direction, i.e., in German from left to right (Breznitz & Bloch, 2012; Breznitz & Nevat, 2006). The aim of this manipulation is to prompt the individual to read at a faster rate than usual as a subjective feeling of time constraint is generated by the text-fading manipulation (Breznitz & Berman, 2003; Nagler, Linkersdörfer, Lonnemann, Hasselhorn, & Lindberg, 2016). As increased reading speed is associated with an increase in reading comprehension (Huemer et al., 2008), the manipulation is supposed to provoke improved reading performance. This notion is tested through the comparison of an experimental group with a control group which receives the same reading material to read in a self-paced manner. Several studies have supported the underlying assumptions, indicating that the text-fading manipulation is associated with faster reading rates and better reading comprehension performance (e.g., Breznitz, 1987, 1997; Nagler et al., 2016; Nagler, Lonnemann, Linkersdörfer, Hasselhorn, & Lindberg, 2014).

### Text-fading training

The promising findings from cross-sectional research designs prompted attempts to implement the text-fading principles into a training procedure aiming at long-term reading fluency improvements. Most text-fading training studies report increased reading rates combined with improved or consistent comprehension performance in pre-post-training comparisons for different orthographies (for a review see Korinth & Nagler, 2021). In general, new reading material is presented for each training trial, providing a direct comparison of comprehension performance during training. Considerable differences and open questions between text-fading training studies can, however, be found regarding the research design, the selected sample, and detectable training effects in standardized tests.

Research sample selection and research design are obviously crucial for any empirical study; however, for intervention studies, they are essential to estimating training efficacy. To capture training effects for children in need of intervention, it is sensible to target samples of reading-impaired participants. Although text-fading training has generally been reported to also be effective for unimpaired elementary school readers (see for example Nagler et al., 2015; Nevo et al., 2016), the training potential should be assessed with regard to samples of impaired readers.

Furthermore, especially long-term effects can only be captured in designs comprising pre-, post-, and follow-up test time points. To our best knowledge, so far only one study has included a pre-post as well as follow-up measurement within a text-fading training approach: Breznitz et al. (2013) reported significant training effects (i.e., increased reading rate and comprehension) from pre- to post-test time point for a group of dyslexic adults receiving a text-fading training compared to a control group of dyslexic adults reading at their self-paced reading routine. In the course of training, the experimental group’s reading performance eventually leveled the performance of a sample of typically reading adults. The reading gains were retained over a 6-month period at follow-up test time point. However, this study tested reading performance of adult readers, so information about long-term effects for elementary school children is still pending.

Questions also remain open regarding whether training effects can be found in standardized tests, and if so, at which reading level (lower- vs. higher-level processing). As reading improvements through training should optimally be transferable to general reading performance, it is advisable to test reading enhancements in an independent, standardized reading test to optimally objectify the training results. Using a standardized test with different reading competency levels (i.e., lower- vs. higher-levels) will furthermore allow to locate the training’s efficacy. One text-fading training study by Nagler et al. (2015) shows significant reading fluency improvements for an experimental group receiving text-fading training compared to a control group reading at their self-paced reading routine in a standardized reading test. Results indicated that reading improvements were attributable to lower as well as higher-level processing, as children in the experimental group improved on word and especially on sentence level reading comprehension. However, this study included typically reading participants, so the transferability of results to a reading-impaired sample has so far not been demonstrated.

## Present study

To address open research questions, the present study focuses on three major aims: (1) With respect to previous findings, showing orthographic knowledge and RAN to be relevant for training outcomes (Berninger et al., 1999; Nelson et al., 2003), we focus on the predictive value of these two reading-related preconditions. We aim at clarifying to what extent orthographic knowledge and RAN are predictors also for the text-fading training approach and can explain variance in comprehension performance during training in both experimental and control groups. We predict that both RAN and orthographic knowledge are significant predictors for comprehension performance during the text-fading training in both training groups. Therefore, children with higher orthographic knowledge and RAN performance are expected to show better reading comprehension performance during text-fading training in both experimental and control groups than peers with lower skill levels. (2) To validate the assumption that training effects from text-fading training are detectable also for children needing intervention, we focus on a sample of German elementary students in grade 3 with reading impairments. In line with previous text-fading training research (e.g., Breznitz et al., 2013; Nagler et al., 2015), we expect to capture potential training effects immediately after the end of the training. For this purpose, we examine the reading rate and comprehension performance in a pre-post comparison of the two training groups. We expect the experimental group to show better reading rate and better comprehension performance after the training compared to the control group. (3) We focus not only on investigating potential, but also lasting training effects associated with text-fading training. Training effects are examined in a standardized reading test on lower and higher reading levels (i.e., word and sentence reading) to capture possible training benefits from the reading intervention. In line with Nagler et al. (2015), we assume that training effects should be detectable at lower as well as higher reading levels. We therefore expect the experimental group to show better reading performance on word as well as sentence level in a standardized reading test after performing the text-fading training in a pre-post comparison. To examine the mere training effects, we will run analyses controlling for individual reading performance before training (i.e., RAN, orthographic knowledge at pre-test point and reading comprehension performance during training). With respect to previous findings by Breznitz et al. (2013), we expect these training effects to be lasting. Hence, predictably, the experimental group will show better reading performance on word and sentence level compared to the control group at a follow-up test time point.

## Materials and methods

### Participants

Following a screening of 13 schools with overall 38 classes, a preselected sample of 484 students participated in the tests. From this preselected sample, 129 children (61 female, mean age: 8.87 years, SD = 0.53) were identified as meeting the criteria for participation in the training study. These children showed significant reading impairments given their reading performance between percentile rank (PR) 0–30 in a standardized reading test (Würzburger Leise-Leseprobe Revision, WLLP-R; [Würzburg Silent Reading Test-Revision], Schneider et al., 2011; mean reading performance: T-score = 26.63, SD = 5.20), while their intelligence quotient (IQ) was classified as average (IQ between ≥ 85 and ≤ 130; mean IQ = 105.80, SD = 15.16) in a standardized test (Zahlen-Verbindungs-Test, ZVT, [A trail making test]; Oswald & Roth, 1987). Parental informed and written consent was obtained for each child. Out of the qualified sample, nine children were not considered for further analyses because they did not attend the training (i.e., the parents withdrew their consent or the children were missing due to personal reasons). Hence, data of 120 German third graders (58 female, mean age: 8.87 years, SD = 0.53) was included for final data analysis. A Monte Carlo power analysis using MPlus 8 (Muthén & Muthén, 1998–2017) indicated that a minimum of 94 participants were required to detect an effect size equal to β = .3, with an alpha set at .05 and power set at .80 (Muthén & Muthén, 2002); hence, the present sample size of N = 120 is presumed to be appropriate for further analyses. Ethical approval was received from the local ethical review board before data collection.

### Design and procedure

**Fig. 1:**
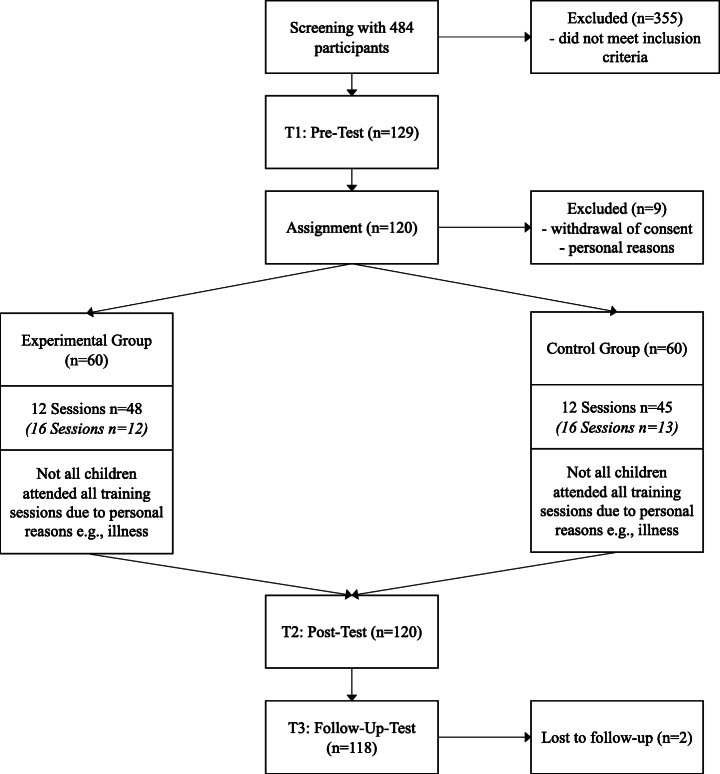
Overview of the number of participants included in the screening, pre-, post-, and follow-up test sessions, as well as training

The applied training consisted of overall 16 training sessions lasting approximately 45 min each, which were offered twice per week, distributed over a period of 8 weeks. In a pre- (T1), post- (T2), and follow-up (T3) design, reading comprehension on word and sentence level was furthermore measured at three test time points. Pre- and post-tests were conducted in a time frame of 3 weeks before and after the training; the follow-up test was realized 6 months after the end of training, also within a time period of 3 weeks. The training and pre-, post-, and follow-up tests were accomplished on the relevant school premises. Children were excused from class for the participation. Participants were randomly assigned to one of two training groups, counterbalanced for gender. The experimental group received training with a text-fading procedure (text-fading group; *n* = 60); the control group was trained without text-fading, thus, at their individual self-paced reading rate (self-paced group; *n* = 60). An initial assessment session (i.e., start session) served to measure the participant’s individual reading rate base level. Here, participants of both groups were instructed to read 30 test items (i.e., sentences with corresponding questions and multiple-choice answer possibilities) at their own self-paced reading rate. The obtained reading rate base level was used for the text-fading group to determine the individually set fading rates (in milliseconds per character), which were applied and adapted during the subsequent text-fading training. The self-paced group continued to read presented text material at their own self-paced reading rate during all training sessions. All participants read 30 sentences per training session. Figure 1 provides an overview of the number of participants included in the screening process, as well as in pre-, post-, and follow-up test sessions and training.

### Test materials

#### Screening tests

In order to identify and recruit children who meet the criteria for participation in the training, a standardized reading test measuring reading (decoding) speed (WLLP-R; Schneider et al., 2011) and a standardized nonverbal intelligence test measuring intellectual abilities via perceptual speed (ZVT; Oswald & Roth, 1987) were applied. Both tests were administered as paper-pencil tests in a classroom setting at the particular schools.

The WLLP-R reading test material for third grade students consists of 7 test sheets, which have to be processed consecutively within 5 min. Each test sheet comprises 20 words accompanied by four pictures, one picture illustrating the target word. In total, 140 items are presented on the consecutive test sheets. Within the time limit, children have to process as many items as possible by choosing the picture matching the word. According to the authors, the WLLP-R shows a high parallel test reliability (third grade: *r* = .93). Conversion tables for individual raw test scores (number of correctly marked test items) to grade- and gender-dependent standardized scores are provided for grade 1 through grade 4.

The ZVT nonverbal intelligence test can be administered in individual and group settings with participants aged between 7 and 80 years. In group settings, as administered in this study, four matrices showing 90 apparently arbitrarily arranged numbers (i.e., 1–90) are consecutively introduced. Within 30 s per matrix, participants have to connect as many numbers in their ascending numerical order as possible. The test’s reliability (retest reliability between *r* = .95 und *r* = .98) is indicated to be mostly independent from age and educational background. Conversion tables for individual raw test scores to age-dependent standardized scores are provided. IQ scores were used for sample selection.

#### Pre-, post-, and follow-up tests

##### Standardized reading comprehension test

To capture possible training effects, a computerized standardized reading comprehension test (Ein Leseverständnistest für Erst- bis Sechstklässler, ELFE 1-6; [A reading comprehension test for first to sixth-graders]; Lenhard & Schneider, 2006) was assessed at T1, T2, and T3 to measure reading performance before and after training as well as after a 6-month intermission period. The two subtests of ELFE 1-6 administered in this study measure reading comprehension at the word and sentence level. Word reading comprehension is measured via 72 items, each item consisting of a picture and four word alternatives with similar graphemes and phonemes. Children were asked to choose the word corresponding to the picture. The number of correctly marked items represents the word reading raw score. Sentence reading comprehension is assessed through a sentence completion task. In this subtest, five possible word alternatives are presented within a total of 28 interrupted sentences. Children have to identify the words completing the sentences. The number of correctly answered items reflects the sentence reading raw score. The two subtests show high internal consistency (word reading: Cronbach’s α = 0.97, sentence reading: α = 0.93). Raw test scores were used for further statistical analyses.

##### Orthographic knowledge

To capture the participants’ orthographic knowledge at the word-specific and general level at T1, we used the item sets applied in a previous study by Zarić et al. (2021). For assessing orthographic knowledge at word-specific level, an orthographic decision task with 30 items (15 real words and 15 pseudohomophones; i.e., words with the same pronunciation as existing words, but incorrectly spelled; e.g., word: *rain*, pseudohomophone: *rane*) was used. Children were instructed to decide whether the presented item was a real word or not. The sum of correctly identified items (raw score) was used for further statistical analyses.

To assess orthographic knowledge at general level, an orthographic choice task with 20 items consisting of two pronounceable pseudo-words was used. In each pair, one of the pseudo-words contained a double consonant in a permitted position (central: e.g., *tammit*, or end position: e.g., *pateff*), while the other contained a double consonant in an illicit position (e.g., *ttokam*). Children were instructed to decide which of the two alternatives presented resembled a real German word more closely. The sum of correctly identified items (raw score) was used for further calculations.

An item analysis regarding orthographic knowledge data was conducted prior to statistical analyses, in order to detect items with a poor level of selectivity and difficulty. For the items used to measure word-specific orthographic knowledge, 14 out of 30 items revealed a poor degree of selectivity (≤ .02) and difficulty (≤ .30 and ≥ .87); hence, they were excluded from further calculations. The sum of the correctly answered remaining 16 items was used for further calculations, showing a sufficient internal consistency (Cronbach’s α = .72). For the items used to measure general orthographic knowledge, 4 out of 20 items revealed a poor degree of selectivity (≤ .10) and difficulty (≥ .87). The remaining 16 items showed a sufficient internal consistency (Cronbach’s α = .77).

##### RAN

To assess participants’ naming speed, a RAN task (based on Denckla & Rudel, 1976) was used. In this task, four matrices in DIN-A3 format were presented, each containing either digits (1, 4, 5, 6, 8), letters (f, k, r, s, t), objects (candle, car, dog, fish, hammer), or colors (blue, green, red, yellow, black). Children were instructed to name the presented stimuli as accurately and as fast as possible. The time required as well as the numbers of errors was measured for each of the four matrices. For each of the four subtests, errors were subtracted from the total score of 50 per subtest, providing the number of correctly named items. Then, the average naming speed (items per second) for each of the four subtests was computed by dividing the total time per subtest by all correctly named items in that subtest for each participant for T1, which was used for further statistical analyses.

#### Training stimuli

A pool of overall 546 items was created for training purposes (6 practice items, 30 items for start session, 30 items for end session, 480 items for the max. of 16 training sessions). Items were self-developed following the reading material used in a previous study by Nagler et al. (2015). Each item comprised a sentence (7–21 words long; mean: 14.07 words) and a corresponding multiple-choice question with four answer possibilities (for training stimuli examples, see Appendix, Table 5). To assure attentive reading of the training items, questions and multiple-choice answers included different phrasing and reasonable distractor items (see Rost & Sparfeldt, 2007). Training stimuli were randomly allocated to the training sessions. Hence, all children read the same reading material within the same session, yet in a randomly assigned order.

### Training paradigm

Participants were trained in a group setting with five to 12 children per session. Training stimuli were presented left-justified in white letters (font: Courier New; letter size: 0.1 height) on a dark background on 15.75-inch laptop computers running the text-fading training paradigm programmed in Psychology software in Python (PsychoPy; Peirce, 2009). Laptop computers were provided by the research team and arranged in an order assuring an undisturbed training procedure. The training started with a start session, followed by max. 16 offered training sessions and ended with an end session. The end session followed the same procedure as the start session. The procedure for the training sessions differed between the two training groups. The number of total completed training sessions varied between children because of absence during the training days, resulting in a range from 4 to 16 completed sessions (*M* = 12.8)^1^.

The start session was identical for both groups and served to determine the initial individual reading rate. After two practice trials, each trial started with the presentation of a single sentence. The children were instructed to read the sentence as fast and as accurately as possible at their own reading pace and press a designated button on the laptop keyboard when finished. The button-press triggered the presentation of the multiple-choice question referring to the respective sentence’s content. The corresponding four answer possibilities comprised the correct answer as well as three incorrect distractors. Children were asked to identify the correct answer and press the according key on the keyboard. Once the children entered their decision, the next trial started. Feedback was not provided. Trials with a reading rate below 50 characters per millisecond were marked as invalid and excluded, as it was considered to be unlikely that the presented sentence was read at such speed. For all remaining correctly answered items, the average reading rate in milliseconds per character and the reading comprehension performance (percent correct) was calculated. Consequent upon the start session, the training sessions were introduced. During the first two training sessions, the children received again two practice trials as well as instruction by the trained examiners. Afterwards, the children proceeded to work independently. Two examiners were present during all training sessions to answer open questions and supervise the course of events.

#### Text-fading group (experimental group)

To ensure that the children were able to fully visually orient, the fading procedure started after a delay of 500 ms. During this time, the sentence was presented in its entirety. The individual average reading rate recorded in the start session was used for the initial text-fading rate in which the reading material disappeared from the screen letter by letter. The multiple-choice questions appeared automatically after the sentence was completely erased. After five trials, the text-fading rate was adjusted depending on the individual’s comprehension performance. If all five items were answered correctly (100% correct), the text-fading rate was accelerated by 2%. At four correct items (80% correct), the text-fading rate was kept constant and with three or less correct items (< 80% correct), the text-fading rate was decelerated by 2%. The last determined text-fading rate of one training session was set as the initial text-fading rate for the following training session.

#### Self-paced group (control group)

The self-paced group read the same sentences and multiple-choice questions as the text-fading group. However, the self-paced group did not receive a text-fading manipulation. Instead, the sentences remained on the screen as long as the children needed to read the sentence and press the designated key to continue. As mentioned above, trials with a reading rate below 50 characters per millisecond were marked as invalid and were excluded. The reading rates (milliseconds per character) and comprehension performance during training (percent correct) were recorded for each trial and each child.

### Statistical analyses

Different statistical approaches were used to address the research questions. For research question 1, a latent variable approach was chosen, for research question 2 a repeated measures analysis of variance (ANOVA) was conducted, and for research question 3 both a repeated measures ANOVA and a latent variable approach were implemented. The analyses were conducted using IBM SPSS (Version 26) and MPlus 8 (Muthén & Muthén, 1998–2017). The latent variables were used to minimize measurement error. To estimate the structural equation model (SEM), the full information maximum likelihood method (ML) was used. To evaluate the goodness of fit for the model, we used the chi-square test statistic and several commonly recommended descriptive measures of model fit (Hu & Bentler, 1998): the standardized root mean squared residual (SRMR), the root mean square error of approximation (RMSEA), the comparative fit index (CFI), and the Tucker-Lewis Index (TLI). Models were evaluated as fitting well when the baseline fit indices (SRMR and RMSEA) were < 0.08 and the incremental fit index (CFI) was ≥ 0.95.

Analyses of missing data lost due to attrition showed that the overall percentage of missing values for the sample was 2.31% with 25 total cases containing missing data (in one to four cells). Little’s missing completely at random (MCAR) test was not significant, *χ*^2^= 161.92, *df* = 177, *p* = .785, suggesting no definitive pattern to the missing data. In order to prevent a loss of power (Graham, Olchowski, & Gilreath, 2007), missing data was addressed via multiple imputation (MI). With this approach, several new data sets were created from original data, in which missing values were replaced by probable values. Overall, 10 imputations were conducted in Mplus. The SEMs were conducted on the imputed and non-imputed data sets. Comparisons yielded no significant differences in the interpretation of results, indicating that magnitude, directions, and statistical significance of effects were consistent. Therefore, all analyses reported are conducted using the imputed data sets to maximize the sample size available.

## Results

As outlined in the following, repeated measures ANOVAs as well as a more sophisticated SEM was chosen as statistical analyses to best answer the three research questions. Table 1 depicts an overview of means and standard deviations of all variables at pre-, post-, and follow-up test time points.

**Table 1:** Means (standard deviation in brackets) of the manifest variables for the self-paced group and the text-fading group at pre-, post-, and follow-up test time points

	T1 (pre-test)		T2 (post-test)		T3 (follow-up test)	
	Control	Intervention	Control	Intervention	Control	Intervention
RAN letters	.73^a^ (.16)	.71^a^ (.19)				
RAN digits	.62^a^ (.14)	.63^a^ (.11)				
RAN colors	1.10^a^ (.30)	1.08^a^ (.23)				
RAN objects	.96^a^ (.17)	.97^a^ (.15)				
Word-specific OK	8.61^b^ (3.27)	8.08^b^ (3.19)				
General OK	12.19^b^ (2.96)	11.87^b^ (3.36)				
Word reading	27.12^c^ (7.85)	28.42^c^ (6.56)	30.48^c^ (8.02)	34.03^c^ (7.81)	35.66^c^ (9.69)	36.53^c^ (8.55)
Sentence reading	9.81^d^ (4.21)	10.73^d^ (3.75)	11.12^d^ (4.27)	12.58^d^ (3.42)	12.89^d^ (4.37)	14.25^d^ (4.05)

*RAN* rapid automatized naming

^a^Average reaction time per correct item in seconds

^b^Raw score (max. 16)

^c^Raw score (max. 72)

^d^Raw score (max. 28)

### Explaining variance in comprehension performance during training

For the first research question, concerning the predictive value of orthographic knowledge and RAN and their contribution of explained variance in comprehension performance during training, a multiple group regression model with latent variables was evaluated with comprehension performance during training as the dependent variable (single indicator latent variable; see Matsunaga, 2008). In this structural equation model (SEM), regression paths were evaluated from both predictors (RAN and orthographic knowledge) to the dependent variable (comprehension performance) while correlations between the predictors were permitted. Four items were used as indicators of RAN, two items for orthographic knowledge. Model identification was achieved by fixing one unstandardized factor loading to 1. Factor loadings and intercepts were set as equal across groups (strong invariance^2^). This multiple group model fitted the data well, *χ*^2^ (32) = 36.79; *p* = .26, CFI = .976, TLI = .968, RMSEA = .050, SRMR = .071, and accounted for a substantial proportion of the variance of comprehension in the self-paced group (*R*^2^ = .38, *p* < .05), but not in the text-fading group (*R*^2^ =.12, *p* = .12). As presented in Table 2, orthographic knowledge was significantly related to comprehension performance during training in the self-paced group (β = .58), whereas no sufficient regression weight was found for the text-fading group (β = .10). A reverse result pattern was found for RAN. While a significant effect was detected for the text-fading group (β = −.31), no sufficient regression weight was revealed for the self-paced group (β = .13). To test whether the standardized regression paths from the predictors on comprehension differ between the two groups, these paths were constrained to be equal across groups and were tested by the Wald test. No significant differences were found for orthographic knowledge, Wald test *χ*^2^(1) = 2.93, *p* = .087, nor for RAN, Wald test *χ*^2^(1) = .71, *p* = .40.

**Table 2:** Standardized effects on comprehension

	Self-paced		Text-fading	
Model variable	B	SE	B	SE
RAN	−.13	.15	−.31*	.16
Orthographic knowledge	.58**	.17	.10	.24
	*R*^2^ = .38		*R*^2^ = .12	

*B* standardized regression coefficient, *SE* standard error, *RAN* rapid automatized naming

**p < .01; *p < .05

Model fit: χ^2^ (32) = 36.79; *p* = .26, CFI = .976, TLI = .968, RMSEA = .050, SRMR = .071

### Potential training effects on reading rate and comprehension performance

**Fig. 2:**
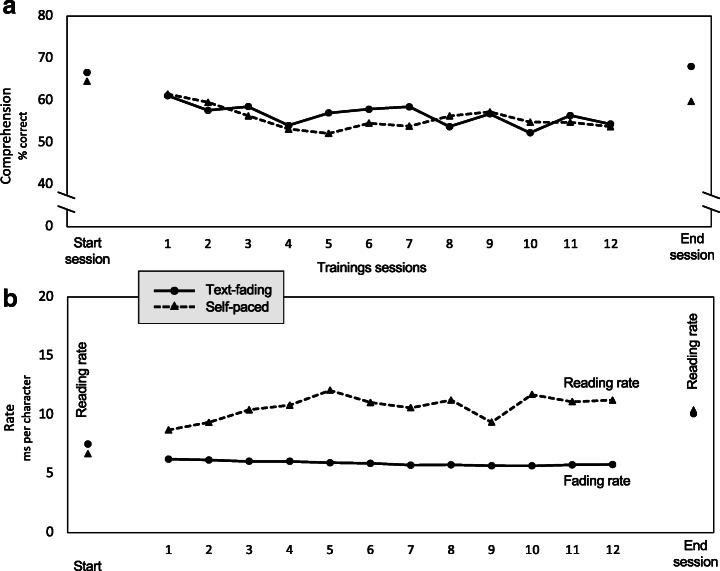
Overview of the text-fading training time course regarding reading comprehension and rate performance for both groups. **a** The time course of reading comprehension performance during training for both groups; **b** the time course of reading rate for the self-paced group and fading speed for the text-fading group; start and end sessions are comparable for all participants

To examine whether there were any significant differences between the self-paced and the text-fading group accountable to the training time course, we carried out ANOVAs with time as a repeated measure variable and group as the between subject variable for reading rate and comprehension as dependent variables. Mean and standard deviation of reading rate and comprehension as a function of time and group can be found in Table 3. The ANOVA revealed that the Group × Time interaction for reading rate was not significant, *F*(1, 118) = .53, *p* = . 47, η^2^ = .004, and there was no significant main effect of group, *F*(1, 118) = .15, *p* = . 70, η^2^ = .001. However, there was a significant main effect of time *F*(1, 118) = 25.31, *p* < .001, η^2^ = .18, indicating that for both groups, reading rate significantly increased during the time course from start to end of the training. For the comprehension measure, a significant interaction effect of Group × Time *F*(1, 118) = 4.10, *p* < .05, η^2^ = .034 was shown, but no significant main effect of group, *F*(1, 118) = 1.81, *p* = .18, η^2^ = .015 or time *F*(1, 118) = 1.84, *p* = .18, η^2^ = .015 was found. Considering the changes in the mean value of the comprehension score (see Table 3), the significant interaction effect of Group × Time is attributable to the decline of the self-paced group at the end of the training. Figure 2 provides an overview of the time course and changes during and after the training in reading rate and comprehension for both text-fading and self-paced group.

**Table 3:** Mean and standard deviation (SD) of reading rate and comprehension as a function of time (start and end of training) and group (self-paced vs. text-fading)

	Start				End			
	Self-paced		Text-fading		Self-paced		Text-fading	
Model variable	M	SD	M	SD	M	SD	M	SD
Reading rate	6.71	2.30	7.47	3.87	10.33	7.76	10.19	6.58
Comprehension in %	64.50	21.68	66.80	21.22	59.72	25.07	67.80	21.04

Reading rate = milliseconds per character; comprehension in % = the percentage of correct answers in the multiple-choice questions after each sentence

### Lasting training effects on word and sentence level

To find out whether the intervention has a positive effect on reading outcomes in a standardized reading test on word and sentence reading levels, firstly, several ANOVAs with time as a repeated measure variable and group as the between subject variable were carried out. We compared (1) T1 and T2 scores controlling for comprehension during training and reading baseline (at T1), as well as (2) T2 and T3 scores for both text-fading and self-paced group controlling for reading performance after training (at T2). The results are presented in Table 4. Regarding the pre-post-test comparison (T1 vs. T2), the analyses revealed a significant interaction of Group × Time for word and sentence reading, indicating that the text-fading group improved significantly more compared to the self-paced group. Regarding the post-follow-up-test comparison (T2 vs. T3), a significant interaction of Group × Time was found for word reading, accountable to the fact that the text-fading group was not able to preserve the performance level and the self-paced group caught up with the text-fading group at T3. For sentence reading, no significant interaction of Time × Group was revealed, indicating that both groups stayed at their performance level in this task 6 months after the text-fading training.

**Table 4:** Results of repeated analysis of variance (ANOVA) for pre- vs. post-test and post- vs. follow-up test

Task measure	Main effects and interactions	*F* (df)	*p*	η^2^
(A) Pre- vs. post-test				
Word reading	Group	6.43 (1,117)	<.05	.05
	Time	83.68 (1,117)	<.05	.42
	Group × time	6.43 (1,117)	<.05	.05
Sentence reading	Group	4.09 (1,116)	<.05	.03
	Time	5.49 (1,116)	<.05	.05
	Group × time	4.09 (1,116)	<.05	.03
(B) Post-test vs. follow-up				
Word reading	Group	3.84 (1,117)	<.05	.03
	Time	48.55 (1,117)	<.05	.29
	Group × time	3.84 (1,117)	<.05	.03
Sentence reading	Group	0.14 (1,116)	.71	.00
	Time	42.84 (1,116)	<.05	.27
	Group × time	0.14 (1,116)	.71	.00

In a second step, we applied SEM to verify the results obtained from the ANOVAs in order to get a more detailed picture of the training effects. Different structural models were calculated to explain variance of word and sentence reading at T2 and T3 (dependent variables) by controlling for the predictors RAN, orthographic knowledge, and comprehension performance during training. All models included a dummy variable representing group condition (0 = self-paced group, 1 = text-fading group) regressed on reading outcomes. To consider initial reading skills and to test possible intervention effects more rigorously, autoregressive effects among the reading outcome (i.e., the effect of a construct on itself measured at a later time) were included in the model. Reading performance at the different test time points (T1, T2, T3) was specified as latent variables, with word and sentence reading as different constructs (single indicator latent variable).

**Fig. 3:**
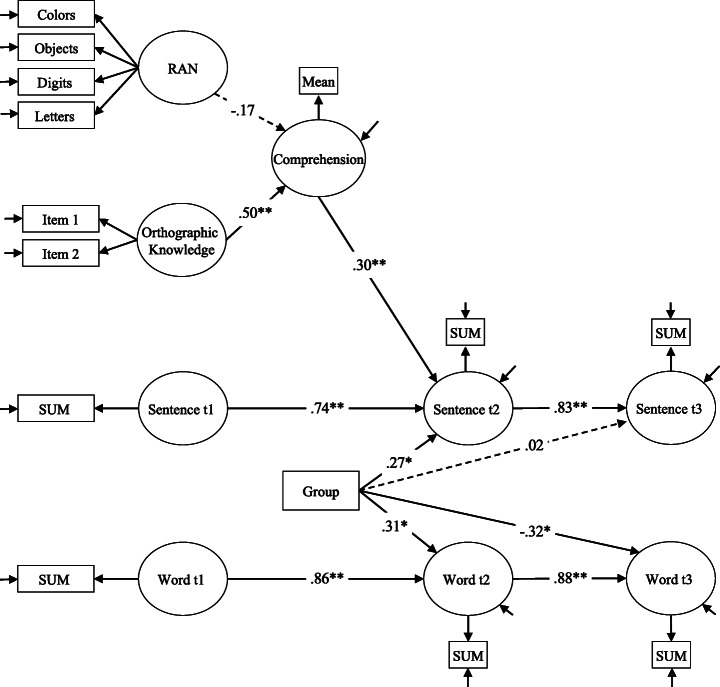
Final structural model showing reading performance on word and sentence level at pre-, post-, and follow-up test being related to group condition (intervention), orthographic knowledge, and RAN. For the sake of clarity, the correlations between the exogenous measures and the correlation between the residuals of word and sentence at T2 and T3 respectively are not depicted. Dashed lines indicate nonsignificant paths. *p < .05; **p < .01

Figure 3 shows the final structural model. This model fitted the data well, *χ*^2^(66) = 99.11, *p* < .001, CFI = .960, TLI = .945, RMSEA = .065, SRMR = .061, and accounted for a substantial proportion of the variance of sentence-reading at T2 (*R*^2^ = .85), and T3 (*R*^2^ = .70), as well as word reading at T2 (*R*^2^ = .77), and T3 (*R*^2^ = .75). As depicted in Fig. 3, significant autoregression effects are found for word (β = .86/.88) and sentence reading (β = .74/.83). Furthermore, sentence-reading at T2 was significantly and positively related to the comprehension performance during training (β = .30). However, most importantly, the group variable explained additional variance in word and sentence reading at T2. The significant positive effects on word and sentence reading at T2 (β = .27/.31) indicate that the text-fading group improved their reading performance stronger from T1 to T2 compared to the self-paced group. A significant negative effect of the group variable on word reading was found at T3, whereas for sentence reading no group effects were shown at T3 test point (see Fig. 3).

## Discussion

The focus of the present study was set on (1) the explanation of variance in comprehension performance during training, as well as on (2) the investigation of potential and (3) also lasting text-fading training effects for a sample of German reading-impaired third graders, after controlling for predictive reading-related variables (i.e., RAN, orthographic knowledge, and comprehension performance during training).

Concerning our first research question, we found orthographic knowledge to be significantly related to comprehension performance during training in the applied structural models for the self-paced group, whereas no sufficient regression weight was found for the text-fading group. For RAN, a reverse result pattern was found, where RAN was identified as a significant predictor for comprehension performance for the text-fading group, but not for the self-paced group. These results indicate that while reading at their own pace, the knowledge about word-specific representations and permitted letter patterns (i.e., orthographic knowledge) supports students’ automatized recognition and reading of single words and enables their processing (e.g., Ehri, 2014). Thus, the better the orthographic knowledge, the more resources are available for understanding the content of the sentences necessary to solve the comprehension task in the present study correctly. However, when reading under time constraint, as provoked in the text-fading condition, the ability to quickly and accurately retrieve the stored orthographic representations from the mental lexicon (reflected in RAN) seems to play a more important role than the mere quality of these orthographic representations. This pattern of results taps into findings from previous research, demonstrating that serial processing is a common mechanism and explanation for the RAN-reading-relationship (e.g., Altani et al., 2018; de Jong, 2011; Georgiou et al., 2013; Protopapas et al., 2018). Following this suggestion, it may be assumed that the text-fading manipulation induces seriality in a task that might otherwise allow for skimming and, more importantly, re-reading. As a consequence, serial processing may have provoked a more fluent (quick and accurate) processing for the text-fading group.

The presented pattern of results concerning predictive values for RAN and orthographic knowledge for comprehension performance during text-fading training is, however, limited to the current data for German reading-impaired children. Whether samples with a normal range of reading variance (i.e., typical reader) would show comparable results still needs to be investigated. Furthermore, as the predictive effect of RAN and orthographic knowledge to reading performance is modulated by the transparency of the orthography, future studies are needed to explore the specific role of both reading predictors in languages with different transparency levels (i.e., transparent, intermediate, and opaque). Additionally, the relevance of other reading precursors (e.g., phonological awareness and verbal working memory) should also be included to better understand the impact and interplay of reading-relevant skills with reading performance during the text-fading training. However, considering the assumption that text-fading training may be an effective tool to increase seriality and consequently enhance reading fluency of children in need for intervention; it seems fruitful to follow the RAN-reading relationship for text-fading training effects in future studies.

The results of the repeated measures ANOVA were pivotal to answer the second research question regarding the potential training effects on reading rate and comprehension performance accountable to the time course of the training. The analyses revealed that the text-fading and self-paced group equally improved their reading rate during the text-fading training; thus, no significant interaction between Group × Time was detected after the training was completed. Regarding the comprehension performance, the analyses revealed a significant interaction between Group × Time. However, this effect is attributable to the decline of the self-paced group comprehension score at the end of the text-fading training. Taken together, the present pattern of results indicates a stable comprehension and reading rate performance during training for both training groups (see Fig. 2). These results are not in line with previous findings showing a significant reading rate improvement in combination with improved or stable comprehension performance in the text-fading group compared to the self-paced group after the text-fading training (for a review see Korinth & Nagler, 2021).

At first sight, these findings seem to be incompatible with our findings regarding our third research question: The results of the repeated measures ANOVA and SEM served to analyze whether or not the text-fading training is effective to produce not only potential but also lasting training effects in German impaired readers on different reading levels measured in a standardized reading test. Significant reading improvements were observable for word and sentence level reading, showing stronger performance increase from T1 to T2 for the text-fading group. These findings seem perplexing at first, as reading rate and reading comprehension did not differ between groups during training, and previous research indicates a connection between improved reading rate and improved reading performance through the text-fading manipulation. However, taking a closer look at the nature of the text-fading manipulation, the results do correspond, suggesting that the reading rate performance and the adaptation of fading rate as an instrument to increase the genuine reading rate in the text-fading group during training may not be the crucial mode of action leading to improved reading performance. It rather supports the notion, as proposed by Korinth et al. (2016) as well as Nagler et al. (2016), that the mere time constraint induced through the text-fading manipulation may be the meaningful mechanism.

With regard to previous findings that fundamental reading processes serve as the basis for word and sentence reading performance (e.g., Wolf & Katzir-Cohen, 2001; Zarić et al., 2021), we propose that the results in our training study can be ascribed to a switch to more efficient reading processes in the text-fading group due to participation in the text-fading training. More precisely, the perceived time constraint may have encouraged the children to switch from basal phonological recoding to more efficient retrieval of complete words or at least parts of words, positively influencing word as well as sentence reading performance. In other words, the feeling of time pressure to read the presented items may have forced the participants in the text-fading group to optimize their reading strategies, possibly engaging in more sophisticated reading strategies, such as direct fact retrieval, while participants in the self-paced group maintained their usual reading strategies. This may have affected the general reading performance, as measurable in a standardized test, resulting in better performance for the text-fading group compared to the self-paced group. These results are in line with previous findings of Nagler et al. (2015) who showed similar results for a sample of non-impaired German elementary school children. Reading-impaired and unimpaired children therefore seem to profit from the fading manipulation in a comparable way, as in both samples participants were able to effectively align their reading behavior on lower- and even on higher-level reading processes resulting in improved reading performance on word and sentence level in a pre-post training comparison.

Finally, the analyses of lasting effects at T3 revealed mixed results, which need to be interpreted cautiously. On the one hand, a negative group effect on word-level was found, indicating that children in the text-fading group stayed at a comparable level at T3 compared to T2, while the self-paced group caught up with the text-fading group. It is possible that the children in the self-paced group made comparable progress in word reading as the text-fading group, however, over a longer period of time, resulting in significant improvement at T3 compared to T2. On the other hand, the absence of negative effects at sentence level indicates that both groups stayed at a comparable reading level at T3 compared to T2. Hence, the reading performance of the text-fading group stayed at a comparable high level at T3, suggesting a preservation of positive training effects for the text-fading group at sentence level observed at T2. Following this suggestion, one interpretation of the maintained high performance at sentence level at T3 test time point for the text-fading group focuses on the training stimuli. It might be that the preservation of training effects was only possible at sentence level because the applied text-fading training contains sentence-based training items; hence, especially sentence reading is trained in this specific intervention approach. However, compared to the present pattern, the long-term effects reported in the study of Breznitz et al. (2013) were clearly more robust. Divergent results between these two studies may be particularly attributed to different samples and training terms. The selected sample in the study by Breznitz et al. (2013) consisted of dyslexic and typically reading adults; hence, the results are not generalizable to children’s reading performance. It has been proposed that adults are more selective and partially more effective regarding the consolidation of memory for specific language skills compared to children (Ferman & Karni, 2010). Therefore, it might be easier for adults to retain improved reading skills acquired during text-fading training than it is for reading-impaired children. Furthermore, Breznitz et al. (2013) conducted more training sessions (24 sessions) than we did in the present study (max. 16 sessions), which might not have been enough to allow for lasting and stable effects in the text-fading group. The validation of lasting effects of the text-fading training approach for reading-impaired children is, hence, not yet clarified and needs further controlled research studies with pre-, post-, and follow-up designs.

### Limitations

The presented data indicates that the text-fading training is effective to produce significant reading improvements for reading-impaired children. However, the underlying mechanisms attributable to the training gains can still not be identified with certainty. Although we were able to identify RAN as relevant predictor for comprehension performance during training for the text-fading group and orthographic knowledge for the self-paced group, we still do not know for sure how training effects emerge and which modes of action trigger the reading improvements. Despite promising results, this study has several limitations: especially methodological and motivational factors will be discussed in the following.

One methodological limitation focuses on the used reading material. Compared to other text-fading training studies, the average reading comprehension performance during training was rather low. Hence, the used reading material might have been too difficult and it was not specifically controlled for age appropriateness. As outlined in a previous study by Nagler and colleagues (2014), the nature of the reading material used during the text-fading procedure directly influences the manipulation’s effectiveness. The authors stressed that only processing of reading material with preconditions for direct lexical retrieval was associated with reading improvements attributable to the manipulation. Considering that the here-included sample showed significant reading impairments, the reading material might have been too challenging to allow for direct lexical access, and in turn, for the full efficacy of the manipulation’s potential.

One further limitation concerns the number of training sessions. As stated above, not all children were able to complete the planned 16 training sessions for various reasons. According to a meta-analysis by Galuschka et al. (2014), reading training approaches should last more than 12 weeks to reveal their full potential and effectiveness. Hence, the implemented max. 8 weeks of training in this study and the comparatively low number of overall completed training sessions (mean: 12.8) might have been insufficient to allow for stable effects in the text-fading group. In order to prevent children from missing a training session, future training studies should be more flexible and provide more time slots during a school day and week, allowing children to catch up on a missing session.

Another methodological limitation is the task used to measure general orthographic knowledge. The task used in this study only measured one aspect of general orthographic knowledge (i.e., the knowledge about permitted/illicit double consonant positions). By including further aspects of general orthographic knowledge (e.g., the knowledge about frequent double consonants), it would be possible to explore its role for reading and reading intervention on a broader scope.

An additional limitation concerns motivational factors. There is an increased risk of motivational problems for children experiencing difficulties in reading (e.g., Alexander-Passe, 2006; Polychroni et al., 2006). Poor readers show lower reading motivation than typical readers (e.g., McGeown et al., 2012; Vaknin-Nusbaum et al., 2018). It is possible that low reading motivation of the sample might have influenced the participants’ endurance during the training, thus affecting the results. Monitoring of motivational state variables as well as motivational support (e.g., through visually more appealing training sessions) could be realized in future text-fading training studies to take care of motivational influence.

## Conclusions

Text-fading training can be acknowledged as an efficient intervention approach for improving reading performance on word as well as sentence level for German elementary school children with reading impairments. However, the detected training gains in a standardized reading test were only explicitly observable in a pre-post comparison; the preservation over a period of 6 months at follow-up test time point was not as clear-cut. Although results indicate a potential maintenance of reading performance on sentence level for the text-fading group, the results need further validation. Several possible explanations of mixed results as well as the accentuation of limitations (e.g., lower consolidation skills of reading-impaired children, difficult nature of reading material, low number of training sessions, motivational effects) might serve to better understand potential influencing factors that need to be considered in applying the text-fading manipulation during training. However, the identification of underlying mechanisms attributable to the training’s effectiveness is still pending and should be further evaluated in order to support children needing intervention.

## Appendix

**Table 5:** Examples of training stimuli in German with an English translation

Sentence in German [English translation]	Word length	Multiple-choice comprehension question [English translation]	Answer alternatives [English translation]
Lisa und die Zwillinge gingen ins Kino. [Lisa and the twins went to the cinema.]	7	Wie viele Personen gingen ins Kino? [How many persons went to the cinema?]	drei [three]^a^; zwei [two]; vier [four]; keine [none]
Wenn Pia groß ist, möchte sie Schriftstellerin werden. [When Pia grows up, she wants to be a writer.]	8	Was möchte Pia machen, wenn sie erwachsen ist? [What does Pia want to do when she grows up?]	Bücher schreiben [write books]^a^; Bilder malen [paint pictures]; Fotos machen [take pictures]; Auto fahren [drive a car]
Der schönste Apfel hing am höchsten Ast des Baums. [The most beautiful apple hung on the highest branch of a tree.]	9	Wo hing der schönste Apfel? [Where did the most beautiful apple hang?]	ganz oben [at the very top]^a^; ganz unten [at the very bottom]; in der Mitte [in the middle]; an der Seite [on the side]
Weil es draußen regnet, können wir heute nicht mehr auf den Spielplatz gehen. [Because it is raining outside, we cannot go to the playground today.]	13	Wie ist es draußen? [What is it like outside?]	nass [wet]^a^; kalt [cold]; heiß [hot]; dunkel [dark]
Mila ist nicht sehr gut im Tennis, deshalb möchte sie an ihrer Technik arbeiten. [Mila is not very good at tennis, that is why she wants to work on her technique.]	14	Was möchte Mila? [What does Mila want to do?]	sich verbessern [improve her skills]^a^; immer gewinnen [win always]; viel spielen [play a lot]; sofort aufhören [stop immediately]
Ben freut sich auf den Sommer, weil es dann wieder frische Beeren und Trauben gibt. [Ben is looking forward to summer because then there are fresh berries and grapes.]	15	Worauf freut sich Ben im Sommer? [What is Ben looking forward to in the summer?]	auf das Obst [to fruits]^a^; auf die Sonne [to sun]; auf den Urlaub [to vacation]; auf das Gemüse [to vegetables]
Emil kennt sich gut mit Dinosauriern aus und ist traurig, dass sie alle schon vor langer Zeit gestorben sind. [Emil is well informed about dinosaurs and is sad that they died a long time ago.]	19	Was sind die Dinosaurier? [What are the dinosaurs?]	ausgestorben [extinct]^a^; gefährlich [dangerous]; bedroht [endangered]; aufgewacht [awoken]
Melanie suchte ihren Schlüssel, den sie sonst immer an die Anrichte im Flur legte, nur heute war er nicht dort. [Melanie searched for her key, which she usually puts on the sideboard in the hallway, but today it was not there.]	20	Was hat Melanie mit dem Schlüssel gemacht? [What did Melanie do with the key?]	ihn verlegt [misplaced it]^a^; ihn verschenkt [gave it away]; ihn abgegeben [passed it on]; ihn gefunden [found it]
Als Isa den Klassenraum verließ, bemerkte sie, dass jemand seinen Block, sein Mäppchen und sein Buch auf dem Tisch vergessen hatte. [When Isa left the classroom, she noticed that someone has forgotten his pad, his pencil case and his book on the desk.]	21	Was hat Isa gefunden? [What did Isa find?]	Schulsachen [school things]^a^; Essen [food]; Zeitschriften [magazines]; Geld [money]

^a^Correct answer

## Footnotes

1 Since the average number of training sessions for the whole sample was 12.8, the comprehension score used for further statistical analyses comprised of the comprehension scores from training sessions 1–12. The number of training sessions did not differ significantly between the text-fading and the self-paced group (*t* test = .381, *p* = .751).

2 Model comparison (scalar against configural): *χ*^2^ (8) = 8.617, *p* = 0.3756.
